# Clinical biomarker profiles reveals gender differences and mortality factors in sepsis

**DOI:** 10.3389/fimmu.2024.1413729

**Published:** 2024-05-21

**Authors:** Zhenglin Chang, Jiancai Lu, Qitai Zhang, Haojie Wu, Zhiman Liang, Xiaocong Pan, Bishan Li, Zhangkai J. Cheng, Baoqing Sun

**Affiliations:** ^1^ State Key Laboratory of Respiratory Disease, Department of Clinical Laboratory, National Center for Respiratory Medicine, National Clinical Research Center for Respiratory Disease, Guangzhou Institute of Respiratory Health, The First Affiliated Hospital of Guangzhou Medical University, Guangzhou, Guangdong, China; ^2^ Guangzhou Laboratory, Guangzhou International Bio Island, Guangzhou, Guangdong, China; ^3^ State Key Laboratory of Respiratory Disease, Department of Blood Transfusion, National Center for Respiratory Medicine, National Clinical Research Center for Respiratory Disease, Guangzhou Institute of Respiratory Health, The First Affiliated Hospital of Guangzhou Medical University, Guangzhou, Guangdong, China

**Keywords:** spesis, gender difference, mortality factors, retrospective analysis, biomarkers

## Abstract

**Background:**

Sepsis is a major contributor to global morbidity and mortality, affecting millions each year. Notwithstanding the decline in sepsis incidence and mortality over decades, gender disparities in sepsis outcomes persist, with research suggesting higher mortality rates in males.

**Methods:**

This retrospective study aims to delineate gender-specific clinical biomarker profiles impacting sepsis progression and mortality by examining sepsis cases and related clinical data from the past three years. Propensity score matching was used to select age-matched healthy controls for comparison.

**Results:**

Among 265 sepsis patients, a significantly higher proportion were male (60.8%, P<0.001). While mortality did not significantly differ by gender, deceased patients were significantly older (mean 69 vs 43 years, P=0.003), more likely to have hypertension (54% vs 25%, P=0.019), and had higher SOFA scores (mean ~10 vs 4, P<0.01) compared to survivors. Principal Component Analysis (PCA) showed clear separation between sepsis patients and healthy controls. 48 serum biomarkers were significantly altered in sepsis, with Triiodothyronine, Apolipoprotein A, and Serum cystatin C having the highest diagnostic value by ROC analysis. Gender-stratified comparisons identified male-specific (e.g. AFP, HDLC) and female-specific (e.g. Rheumatoid factor, Interleukin-6) diagnostic biomarkers. Deceased patients significantly differed from survivors, with 22 differentially expressed markers; Antithrombin, Prealbumin, HDL cholesterol, Urea nitrogen and Hydroxybutyrate had the highest diagnostic efficiency for mortality.

**Conclusion:**

These findings enhance our understanding of gender disparities in sepsis and may guide future therapeutic strategies. Further research is warranted to validate these biomarker profiles and investigate the molecular mechanisms underlying these gender differences in sepsis outcomes.

## Introduction

Sepsis, a syndrome characterized by physiological, pathological, and biochemical abnormalities induced by infection, represents a significant public health issue (1). Bacterial infections are primarily considered the cause of sepsis. However, fungal and viral infections, such as AIDS and rickettsiosis, can also lead to sepsis in some cases (2). These causes can weaken the immune system’s resistance to infection, leading to a range of symptoms. The primary symptom of sepsis is a systemic inflammatory response, often accompanied by the failure of some organs (3). If not treated timely, sepsis can escalate into a more severe form—septic shock, associated with higher mortality rates (2). Once diagnosed with sepsis, patients generally require immediate treatment to combat the infection and reverse life-threatening organ dysfunction. Treatment of critical organs often necessitates admission to Intensive Care Units (ICU) or medical wards, possibly requiring long-term use of these expensive healthcare facilities (4). Thus, sepsis imposes a significant economic burden on global healthcare systems. In 2017, an estimated 48.9 million cases of sepsis were reported worldwide, with 11 million sepsis-related deaths, accounting for 19.7% of all global deaths. From 1990 to 2017, the age-standardized incidence rate of sepsis decreased by 37.0%, and the mortality rate decreased by 52.8% (5). Despite this, the burden of sepsis remains, continuing to be one of the leading causes of hospital mortality worldwide, especially in low and middle-income countries (2).

Most research on the risk factors for death from sepsis has focused on susceptibility to infection and organ dysfunction. Besides recognized risk factors like age, immunosuppressive diseases, and chronic conditions such as diabetes and chronic obstructive pulmonary disease, gender may also influence sepsis outcomes. Over the past decade, numerous clinical and epidemiological studies have investigated the impact of gender on sepsis (6, 7). Some epidemiological studies found that the incidence of sepsis was lower in women compared to men (8, 9). However, the evidence on gender-dependent clinical outcomes in sepsis from many observational clinical studies is inconsistent, with no clear data on how gender affects outcomes once sepsis occurs (7–11). Additionally, while immune response dysregulation and organ failure have been identified as primary factors of death, the varied clinical manifestations and outcomes among infectious patients suggest differences in pathology (12). The inability to identify specific immune responses and the complex pathophysiology of organ dysfunction leads to poor prognosis and high mortality rates in sepsis (13). Numerous studies have successfully identified new functions of related signaling pathways, but few have endeavored to elucidate the primary gender- and mortality- related clinical biomarker profiles of sepsis progression (14).

In conclusion, the underlying clinical factors contributing to gender disparities in sepsis, as well as the clinical features culminating in mortality, remain obscure. This research aims to shed light on these areas by scrutinizing sepsis cases and pertinent clinical data collected over the previous three years at our institution. By employing propensity score matching, we have selected age- and gender-matched healthy controls from routine health screening participants for comparative analysis. Our objective is to delineate the distinctive clinical biomarker profiles associated with gender variations in the course of sepsis, and to investigate the determinants of these gender-based differences as well as the specific molecular determinants of mortality in septic patients.

## Methods

### Patient recruitment

The selection criteria for sepsis patients in this study were as follows: (1) Referring to the 2016 “Third International Consensus Definitions for Sepsis and Septic Shock” (1); (2) Complete clinical data. The exclusion criteria for sepsis patients were: (1) Patients with malignant tumors; (2) Patients with hematologic diseases; (3) Patients with severe liver diseases and severe hemorrhage; (4) Patients recently treated with immunosuppressants; (5) Patients with unclear diagnosis and incomplete clinical laboratory records. The inclusion criteria for the healthy control group used for comparison were as follows. Firstly, healthy individuals from recent medical examinations were selected based on the following requirements: (1) Healthy status, no comorbid diseases; (2) No infections requiring systemic antibiotic treatment in the past month; (3) Each examinee had more than 15 test items checked. The study was deemed exempt from review by the Institutional Review Board of the First Affiliated Hospital of Guangzhou Medical University.

### Data collection

Clinical information were collected by researchers. The clinical information includes sex, age, smoking history, history of drinking, and ABO blood group. Specifically, the systolic pressure (SP), diastolic pressure (DP), mean arterial pressure (MAP), and heart rate (HR) values were collected either throughout the hospital stay or at discharge. Other data collected included respiration, temperature, duration of hospital stay, sequential organ failure assessment score (SOFA score), height, weight, BMI, source of infection, death, hypertension, diabetes, surgical history, and whether surgery was performed.

### Propensity score matching

Propensity Score Matching (PSM) is a statistical analysis method used to address potential selection bias in observational data. PSM aims to create a propensity score, which is then used to match or pair individuals in different groups within a study (15). This approach helps to reduce potential confounders, making comparisons more comparable (15). In this study, gender and age were used as covariates to match a healthy control group at a 1:2 ratio, meaning one patient was matched with two healthy individuals undergoing medical check-ups.

### Laboratory testing of human blood

The serum test indicators were obtained by centrifuging blood samples at 3000 revolutions per minute, taking the upper layer of serum, and analyzing it using an automatic biochemistry analyzer to obtain data for 113 test items. For patients with sepsis, the serum samples were collected either at discharge or during hospitalization. All serum samples were detected by the Department of Clinical Laboratory, the First Affiliated Hospital of Guangzhou Medical University.

### Differential analysis

Differential analysis is commonly used to determine if there are significant differences between two or more datasets (16–18). In this study, statistical significance (usually represented by the P-value) and effect size (such as the log-transformed fold change, i.e., log fold change) are used as criteria for selecting differential indicators. The threshold for P-value is set at 0.05 with a fold change value set at 1.5.

### Receiver operating characteristic

The Receiver Operating Characteristics (ROC) curve is a commonly used tool for evaluating the classification performance at different threshold levels. The Area Under the Curve (AUC) of the ROC curve further aggregates the average performance of a given classifier by calculating the area under the curve. This metric is particularly valuable because it is not dependent on label distribution (19). It is commonly employed in various applications, including disease prediction (20). In this study, an AUC greater than 0.70 was used as a criterion to select indicators with high diagnostic efficacy.

### Statistical analyses

All statistical analyses were conducted using R (version 4.0.2) and IBM SPSS Statistics (version 25.0). The ‘limma’ package in R was used for differential analysis. Chi-square tests were applied to detect differences in categorical variables, which were reported as percentages (%). Statistical significance of continuous variables between two groups was estimated through Student’s T-test or Mann-Whitney-Wilcoxon test, with criteria for differential indicators being FDR<0.05 and an absolute log2(Fold change)>1. ROC analysis was employed to assess the diagnostic efficiency of diagnostic indicators, using the AUC value to select the top 20 diagnostic indicators, for which the R package ‘pROC’ was utilized. The ‘ggplot2’ package in R was used for graphical plotting, and Adobe Illustrator was employed for color and layout optimization, as previously described (21). For handling null and missing values, this study identified both using R, initially filling them using the mean value of each test item. Subsequently, if an entire column was null, it was removed using R.

## Results

### Demographics and clinical characteristics

We collected clinical data from 265 patients diagnosed with sepsis over the past three years. Among them, 104 were female, accounting for 39.2%, and 161 were male, accounting for 60.8%. The proportion of male patients was significantly higher than that of female patients (P<0.001). Regarding age distribution, there was no significant difference between genders (P=0.525). In terms of mortality, a total of 10 patients died, including 2 females and 8 males, but this difference was not significant (P=0.13). Additionally, significant gender differences were observed in terms of unhealthy lifestyle habits, such as history of smoking and drinking (**Table 1**).

**Table 1:** Characteristics of 265 patients (161 males and 104 females) according to gender.

	level	female	male	p
N		104	161	
Sex (%)	female	104 (100.0)	0 (0.0)	<0.001
	male	0 (0.0)	161 (100.0)	
Age (mean (SD))		42.69 (32.56)	45.18 (30.12)	0.525
smoking history (mean (SD))		0.03 (0.17)	0.24 (0.43)	<0.001
history of drinking (mean (SD))		0.01 (0.10)	0.07 (0.26)	0.017
SP (mean (SD))		107.05 (24.60)	108.50 (21.19)	0.623
DP (mean (SD))		65.95 (14.13)	66.48 (14.11)	0.772
MAP (mean (SD))		79.67 (16.20)	80.49 (15.09)	0.686
Respiration (mean (SD))		23.72 (5.28)	24.01 (5.33)	0.682
Temperature (mean (SD))		37.21 (1.09)	37.14 (1.04)	0.625
HR (mean (SD))		107.37 (25.34)	105.27 (27.09)	0.541
Hospital stay duration (mean (SD))		15.24 (19.08)	14.66 (18.27)	0.821
SOFA (mean (SD))		3.78 (4.73)	4.60 (5.09)	0.204
qSOFA (mean (SD))		0.79 (0.72)	0.81 (0.68)	0.768
Hight (mean (SD))		136.16 (29.35)	144.05 (36.76)	0.096
Weight (mean (SD))		41.99 (25.12)	46.62 (24.18)	0.177
BMI (mean (SD))		20.04 (7.34)	20.17 (4.89)	0.874
Death (%)	Alive	102 (98.1)	150 (93.2)	0.13
	Deceased	2 (1.9)	11 (6.8)	
Hypertension (mean (SD))		0.24 (0.43)	0.27 (0.45)	0.553
Diabetes (mean (SD))		0.21 (0.41)	0.14 (0.35)	0.147
Surgical history (mean (SD))		0.32 (0.47)	0.32 (0.47)	0.993
Whether surgery (mean (SD))		0.19 (0.40)	0.17 (0.37)	0.61
ABO (%)		65 (62.5)	109 (67.7)	0.299
	A	11 (10.6)	19 (11.8)	
	AB	2 (1.9)	0 (0.0)	
	B	8 (7.7)	14 (8.7)	
	O	18 (17.3)	19 (11.8)	

When comparing deceased patients with those who survived, we found significant differences in age from the baseline data (P=0.003); the average age of deceased patients was 69 years, while that of surviving patients was 43 years. In terms of hypertension, the proportion of deceased patients with the condition was 54%, significantly higher than the 25% in surviving patients (P=0.019). Moreover, the average SOFA score for deceased patients is around 10, significantly higher than that of surviving patients (P < 0.01) (**Table 2**).

**Table 2:** Characteristics of 265 patients (252 survivors and 13 deceased) according to death.

	level	Alive	Deceased	p
n		252	13	
Sex (%)	female	102 (40.5)	2 (15.4)	0.13
	male	150 (59.5)	11 (84.6)	
Age (mean (SD))		42.91 (31.13)	69.31 (15.70)	0.003
smoking history (mean (SD))		0.15 (0.36)	0.31 (0.48)	0.132
history of drinking (mean (SD))		0.04 (0.20)	0.15 (0.38)	0.073
SP (mean (SD))		107.99 (22.30)	106.54 (28.29)	0.822
DP (mean (SD))		66.54 (13.96)	61.38 (16.05)	0.2
MAP (mean (SD))		80.37 (15.29)	76.44 (19.52)	0.375
Respiration (mean (SD))		23.88 (5.38)	24.15 (3.91)	0.856
Temperature (mean (SD))		37.17 (1.06)	37.06 (1.11)	0.713
HR (mean (SD))		106.32 (26.59)	102.00 (22.68)	0.566
hospital stay duration (mean (SD))		15.06 (18.93)	12.23 (11.35)	0.595
SOFA (mean (SD))		3.96 (4.66)	9.85 (6.78)	<0.001
qSOFA (mean (SD))		0.79 (0.70)	1.00 (0.71)	0.296
Hight (mean (SD))		139.83 (34.54)	162.30 (9.97)	0.042
Weight (mean (SD))		44.19 (24.94)	56.70 (11.38)	0.117
BMI (mean (SD))		20.03 (5.97)	21.92 (6.45)	0.331
Hypertension (mean (SD))		0.25 (0.43)	0.54 (0.52)	0.019
Diabetes (mean (SD))		0.16 (0.37)	0.31 (0.48)	0.176
Surgical history (mean (SD))		0.32 (0.47)	0.23 (0.44)	0.495
Whether surgery (mean (SD))		0.18 (0.39)	0.08 (0.28)	0.333
ABO (%)		166 (65.9)	8 (61.5)	0.564
	A	27 (10.7)	3 (23.1)	
	AB	2 (0.8)	0 (0.0)	
	B	22 (8.7)	0 (0.0)	
	O	35 (13.9)	2 (15.4)	

**Figure 1:**
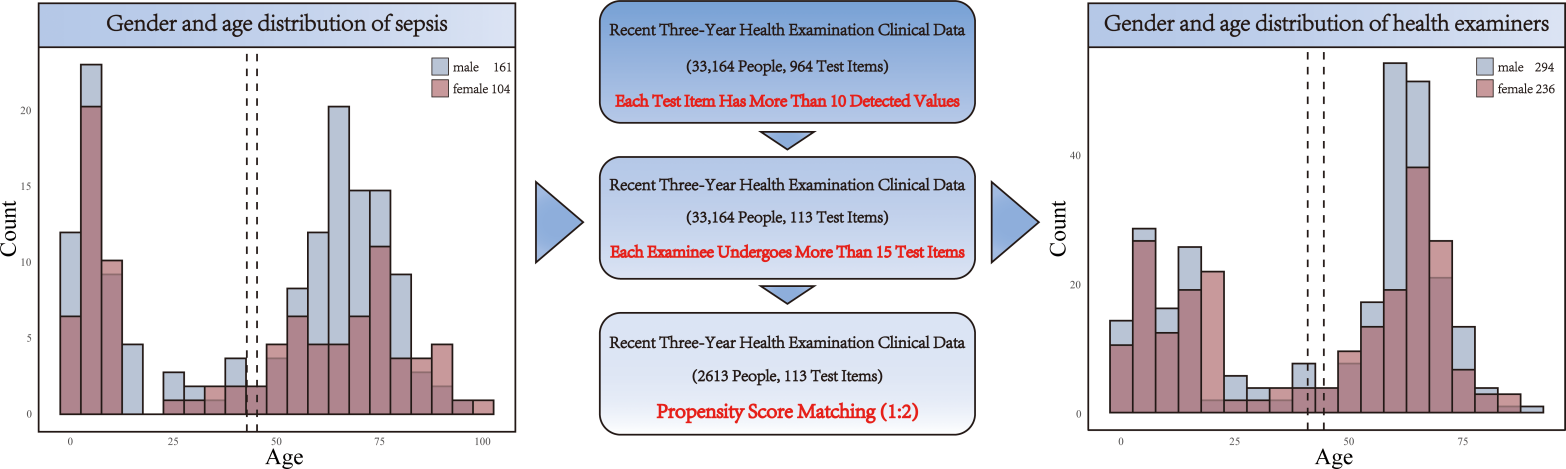
Age and gender distribution of patients and healthy individuals.

We next collected data from 33,164 healthy individuals who underwent physical examinations (**Figure 1**). After screening and 1:2 propensity score matching, we identified a cohort of 530 healthy individuals for comparison in the subsequent analysis (**Supplementary Table S1**). The matching process was designed to ensure that the control group closely resembled the patient group in terms of age distribution. The left histogram in our analysis represents sepsis patients, showing a specific age distribution pattern. Conversely, the right histogram displays the age distribution for the healthy control group, derived through the matching process. The comparison between these two groups illustrates the effectiveness of the matching strategy in selecting a control population with similar age characteristics to the sepsis patients. This approach allows for a more accurate assessment of the impact of sepsis on patients, free from confounding age-related factors (**Figure 1**).

### Comparisons between health control and spesis

**Figure 2:**
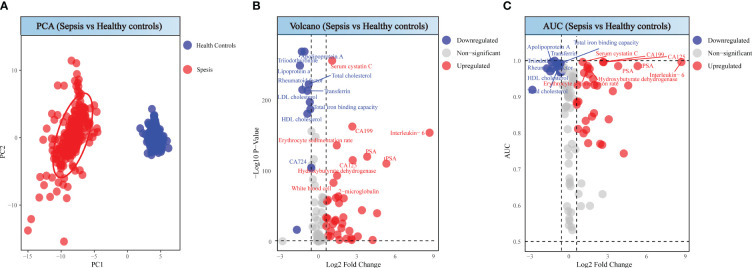
Analysis between sepsis patients and healthy individuals. **(A)** PCA score of sepsis patients and healthy individuals. **(B)** Volcano plot of sepsis patients and healthy individuals. **(C)** ROC analysis for sepsis patients and healthy individuals.

Through Principal Component Analysis, a clear segregation between sepsis and health controls was observed (**Figure 2A**), suggesting the uniquely variability in sepsis patients. Volcano plots highlighted 48 significantly altered serum biomarkers between two groups, based on filtering criteria of p < 0.05 and log2FC <- 0.584962501 or log2FC > 0.584962501, in which values of 37 detection indicators significantly increased, while values of 11 indicators significantly decreased (**Figure 2B**). To assess the diagnostic value of each serum biomarker, we conducted ROC analysis. Combining AUC values (**Figure 2C**), the top 20 diagnostic efficacy indicators with distinctiveness are Triiodothyronine, Apolipoprotein A, Serum cystatin C, Total cholesterol, Rheumatoid factor, Transferrin, Total iron binding capacity, HDL cholesterol, CA199, Interleukin- 6, Erythrocyte sedimentation rate, PSA, CA125, tPSA and Hydroxybutyrate dehydrogenase.

### Comparison of patient and healthy individuals according to sex

**Figure 3:**
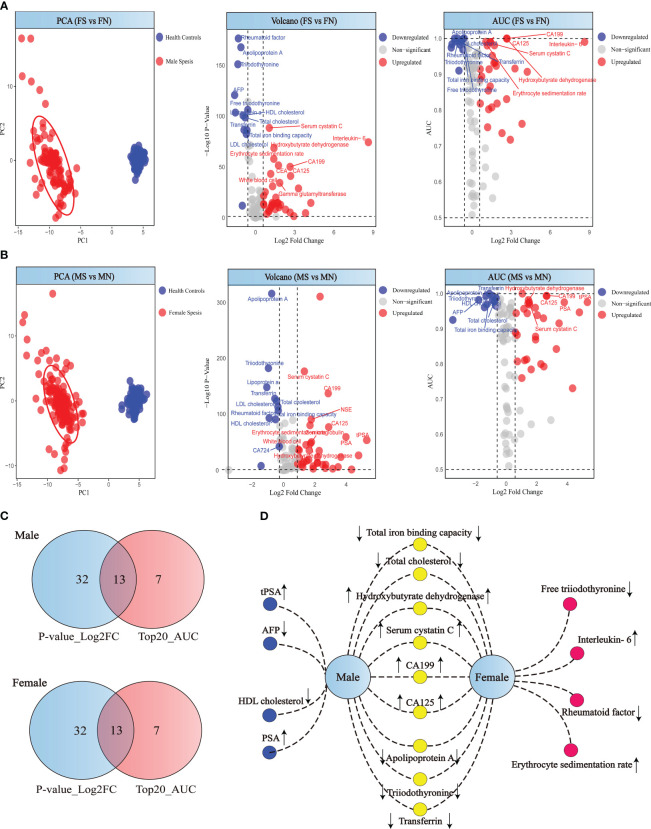
Analysis of patient and healthy individuals according to sex. **(A)** PCA score, volcano plot and ROC analysis for female patient and healthy individuals. **(B)** PCA score, volcano plot and ROC analysis for male patient and healthy individuals. **(C)** Differential indicators and top 20 diagnostic efficiency indicators Venn diagram of the male and female. **(D)** Gender-specific biomarkers in patients. Yellow circles represent biomarkers with high diagnostic efficacy shared by both male and female sepsis patients. Red circles indicate biomarkers uniquely significant for diagnosing sepsis in female patients, while blue circles denote those specifically relevant for male sepsis patients.

Our comparative analysis revealed distinct biomarker profiles when contrasting sepsis patients with healthy individuals, differentiated by sex. Principal Component Analysis (PCA) demonstrates clear segregation between patients and healthy controls for both genders (**Figures 3A, B**). A rigorous selection criterion (p < 0.05 and |log2FC| > 0.58) identified 45 differentially expressed markers in both male and female cohorts. We next intersected the differentially expressed markers with those ranking in the top 20 for diagnostic efficacy, revealing gender-specific and shared biomarkers with high diagnostic value through a network of connections (**Figures 3C, D**). In males, biomarkers such as AFP, HDL cholesterol, tPSA, and PSA stood out with high diagnostic value, with AFP uniquely showing a downward trend. In females, Free triiodothyronine, Rheumatoid factor, Interleukin-6, and Erythrocyte sedimentation rate emerged as significant, with Interleukin-6 and Erythrocyte sedimentation rate notably increased, and the other markers decreased, highlighting potential gender-specific pathways in sepsis pathophysiology (**Figures 3C, D**).

### Comparison of patient according to death

**Figure 4:**
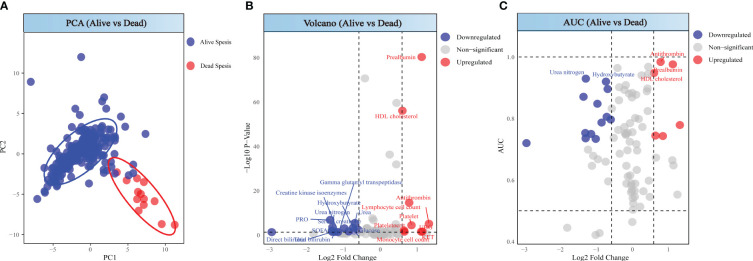
Analysis between alive sepsis patients and dead sepsis patients. **(A)** PCA score of alive sepsis patients and dead sepsis patients. **(B)** Volcano plot of alive sepsis patients and dead sepsis patients. **(C)** ROC analysis for alive sepsis patients and dead sepsis patients.

Patients in the death group showed significant differences compared to those in the survival group (**Figure 4A**). Through differential analysis, with the selection criteria of p < 0.05 and log2FC < -0.58 or log2FC > 0.58, we found 22 differentially expressed markers in the death, in which values of 13 detection indicators significantly increased, while values of 9 indicators significantly decreased (**Figure 4B**). Combining the AUC values, the indicators with high diagnostic efficiency are Antithrombin, Prealbumin, HDL cholesterol, Urea nitrogen and Hydroxybutyrate. Among these, the values of the first three markers are increasing, while those of the latter two are decreasing (**Figure 4C**).

## Discussion

After conducting a retrospective analysis of patients over three years, significant differences were noted in unhealthy lifestyle habits (smoking and drinking) between male and female patients, with deceased patients having a higher proportion of suffering from hypertension. Analysis revealed that biomarkers for male patients included AFP, HDL cholesterol, tPSA, and PSA, while those for females were Free triiodothyronine, Rheumatoid factor, Interleukin-6, and Erythrocyte sedimentation rate. Significant difference indicators between deceased and surviving patients were Antithrombin, Prealbumin, HDL cholesterol, Urea nitrogen, and Hydroxybutyrate. These indicators reflect the specific clinical manifestations of gender differences in the progression of sepsis and may provide direction for exploring the driving factors behind gender differences in sepsis and the specific molecular factors leading to patient death.

We found that male patients showed significant differences from females in terms of history of alcohol consumption and smoking. There is currently no research evidence that smoking directly leads to sepsis, while existing studies have found that long-term ethanol consumption increases the risk of sepsis (22), possibly explaining the higher number of male patients over the three years. Current research focuses on the impact of smoking and alcohol abuse on the outcome of sepsis. While the effect of smoking on immune function is well documented (23), the overall impact of smoking on the clinical outcomes of sepsis is not clear, with some (24, 25) but not all (24, 26, 27) reports indicating adverse effects of smoking on sepsis outcomes. Alcohol consumption affects the immune system’s response, regulating the function of most immune cells, thereby compromising effective immune responses and leading to decreased resistance to infection (28, 29); chronic alcohol abuse has been shown to increase the mortality rate of sepsis (22), with an increased mortality rate of sepsis observed in animal models of sepsis after excessive ethanol consumption (29, 30). Sepsis patients with a history of chronic alcohol abuse significantly increased the likelihood of respiratory impairment and had a higher risk of developing acute respiratory distress syndrome, a leading cause of death, possibly through altering lung glutathione homeostasis (28); moreover, alcohol abuse easily leads to alcohol-related liver disease (29), and sepsis is one of the most common complications and causes of death in patients with alcohol-related liver disease (31), possibly related to impaired formation of neutrophil extracellular traps and weakened phagocytosis (32). Our study found certain differences in these aspects between deceased and surviving patients, but not significant enough to meet p<0.05.

High-density lipoprotein cholesterol (HDL-C) represents the level of high-density lipoproteins (HDL) in the blood, playing a crucial role in immune responses and regulating endothelial function (33, 34). Its anti-inflammatory action might slow the progression of sepsis (35). Current research shows that HDL levels significantly decrease in sepsis patients (33, 36), a result matching ours, possibly because sepsis triggers a systemic inflammatory response, leading to the release of inflammatory cytokines like tumor necrosis factor α (TNF-α) and interleukin-6 (IL-6) (34), which inversely correlate with HDL (35), suggesting that inflammatory factors may indirectly reduce HDL synthesis by inhibiting hepatic lipid metabolism. In our study, HDL-C was a differential diagnostic indicator of higher efficacy in males than females, showing a more significant decrease in male patients. Typically, adult women have higher HDL-C levels than men (37); thus, female HDL-C levels might maintain a functional quantity after being affected by sepsis. Furthermore, the prevalence of smoking among male patients, known to reduce HDL-C levels (38, 39), may contribute to the greater differences observed in male patients.

No literature currently indicates normal IL-6 levels vary by gender; a meta-analysis showed healthy donors’ IL-6 levels ranged from 0 to 43.5 pg/ml (40). IL-6 is considered a biomarker with high diagnostic and prognostic value in sepsis (41–43), quickly rising to peak levels early in sepsis (41). This matches our findings of significantly increased IL-6 expression in female patients; male patients showed no significant change, possibly due to insufficient data. However, smoking has been proven to increase the production of inflammatory cytokines associated with hepatocellular damage, including IL-6 (44), suggesting IL-6 levels might be higher and more variable in male patients, a hypothesis that requires further exploration. The potential mechanism for IL-6 increase involves endotoxin release from the cell walls of infecting bacteria into the bloodstream, binding with Toll-like receptor 4 (TLR4) on immune cell surfaces, triggering a series of signal transduction pathways that ultimately activate transcription factors like nuclear factor κB (NF-κB), promoting the expression of various inflammation-related genes, including IL-6. Moreover, IL-6 activates two major intracellular signaling pathways, JAK-STAT3 and MAPK, by binding with its soluble IL-6 receptor (sIL-6R) and gp130 complex, which can induce IL-6 gene transcription.

Deceased patients showed a significant difference in hypertension prevalence, a poor prognostic factor for acute kidney injury (AKI) development in sepsis patients (45), hinting at increased mortality risk for hypertensive patients in sepsis. Among the five biomarkers identified through comparing deceased and surviving patients, HDL cholesterol was related to hypertension. Research found that hypertensive patients commonly have lower levels of HDL-C (46), contrasting with our results where HDL-C levels unexpectedly rose in deceased patient post-severe sepsis, possibly due to mechanisms causing an increase in levels during exacerbated patient conditions, even though studies also indicated significantly lower HDL-C levels in non-survivors compared to survivors and no significant difference between the two groups (47). Considering the small number of deceased patients, further analysis with a larger sample size is needed for verification.

Our findings also revealed that levels of apolipoprotein A, total cholesterol, and transferrin were significantly decreased in sepsis patients, while levels of cystatin C, rheumatoid factor, TIBC, CA19-9, ESR, PSA, CA125, tPSA, and hydroxybutyrate dehydrogenase were markedly elevated. These changes reflect the complex pathophysiological alterations induced by sepsis. For instance, the decreased levels of apolipoprotein A and total cholesterol suggest a perturbation in lipid metabolism, which may contribute to the increased susceptibility to infections and organ dysfunction in sepsis (48). Elevated cystatin C levels indicate reduced renal function, a common complication in sepsis (49). The alterations in transferrin and TIBC suggest a disturbance in iron homeostasis, which may exacerbate oxidative stress and impair immune function (50). Furthermore, the elevated levels of multiple tumor markers, such as CA19-9, PSA, and CA125, indicate that sepsis may increase the susceptibility to tumors. Lastly, a systematic review and meta-analysis with meta-regression revealed that serum hydroxybutyrate dehydrogenase levels were significantly correlated with markers of inflammation, sepsis, liver injury, non-specific tissue damage, myocardial injury, and renal function (51). These findings underscore the extensive impact of sepsis on multiple organs and systems, and highlight the potential of these markers in guiding sepsis diagnosis and treatment.

In concluding our study, we have unveiled significant insights into the biomarker profiles of sepsis patients, suggesting that a biomarker panel could serve as a superior diagnostic tool compared to single markers. The complexity of sepsis necessitates such a multifaceted diagnostic approach, which promises to refine diagnostic accuracy, aid in sepsis severity classification, and potentially inform treatment choices. We advocate for the integration of these biomarkers into current clinical practices to enhance a personalized approach to sepsis treatment. However, we must acknowledge certain limitations. Our research did not examine the impact of sex hormones or menstrual cycles, which may affect the applicability of our results across a broader demographic. Additionally, we did not identify the pathogens responsible for sepsis nor investigate gender-based variations in pathogen type and sepsis severity. These elements are crucial to understanding sepsis’s intricacies and the absence of this data could narrow the scope of our findings.

## Data availability statement

The original contributions presented in the study are included in the article/**Supplementary Material**. Further inquiries can be directed to the corresponding author.

## Ethics statement

Ethical approval was not required for the study involving humans in accordance with the local legislation and institutional requirements. Written informed consent to participate in this study was not required from the participants or the participants’ legal guardians/next of kin in accordance with the national legislation and the institutional requirements.

## Author contributions

ZC: Conceptualization, Data curation, Writing – original draft. JL: Investigation, Writing – review & editing. QZ: Validation, Writing – original draft. HW: Validation, Writing – original draft. ZL: Validation, Writing – original draft. XP: Conceptualization, Writing – review & editing. BL: Methodology, Writing – original draft. ZC: Methodology, Writing – review & editing. BS: Project administration, Supervision, Writing – review & editing.
